# Phase angle as a prognostic factor for postoperative outcomes in major abdominal surgery: a single-center prospective observational study

**DOI:** 10.1007/s00540-025-03526-6

**Published:** 2025-06-13

**Authors:** Yu Jeong Bang, Dawoon Jeong, Ji Hye Kwon, Yang Jin Park, Jeong‑Jin Min

**Affiliations:** 1 Department of Anesthesiology and Pain Medicine, Samsung Medical Center, Sungkyunkwan University School of Medicine, Seoul, Korea 81 Irwon-Ro, Gangnam-Gu,; 2 Department of Anesthesiology and Pain Medicine, CHA Ilsan Medical Center, CHA University School of Medicine, Goyang, Korea; 3 Division of Vascular Surgery, Department of Surgery, Samsung Medical Center, Sungkyunkwan University School of Medicine, Seoul, Korea

**Keywords:** Bioelectrical impedance analysis, Frailty, Major abdominal surgery, Phase angle, Postoperative complications, Prognostic nutritional index

## Abstract

**Purpose:**

Phase angle (PA), derived from bioelectrical impedance-analysis (BIA) has emerged as a reliable marker predicting clinical outcomes. This prospective observational study investigated the association between PA and a composite in-hospital outcome in major abdominal surgery.

**Methods:**

Each patient underwent BIA before surgery (PA_pre_), immediately postoperatively (PA_post_), and 1 day postoperatively (PA_POD1_). Specific assessment for frailty and nutrition status was performed before surgery. Patient outcomes were assessed using a composite adverse outcome comprising death, myocardial infarction, revascularization, stroke, hemodynamic instability, acute kidney injury, pulmonary complications, delirium, ileus, and surgical complications during hospitalization. One-year complication, including all-cause mortality, myocardial infarction, stroke, surgical complications, and readmission after discharge within the year were also assessed.

**Results:**

A total of 122 adults who underwent major abdominal surgery were enrolled from July 2019 and April 2021. Twenty-three patients (53.5%) in the lower PA group (PA < 5) experienced in-hospital complications compared to 38 patients (34.2%) in the higher PA group (PA ≥ 5) (relative risk, 1.6; 95% confidence interval [CI], 1.0 to 2.4; *p* = 0.038). PA_pre_ was significantly associated with in-hospital complications (odds ratio, 0.491; 95% CI, 0.279 to 0.862; *p* < 0.001). Patients with lower PA_pre_ had a higher degree of frailty, and poor nutritional status. However, PA_pre_ was not significantly associated with 1-year composite complications.

**Conclusion:**

Low PA_pre_ was associated with adverse postoperative outcomes after major abdominal surgery. PA can be a reliable prognostic factor to predict in-hospital complications in patients undergoing major abdominal surgery, serving as an alternative surrogate to frailty indices and nutritional markers.

**Trial registration:** Clinical Research Information Services of the Republic of Korea (CRIS identifier: KCT0004160).

**Supplementary Information:**

The online version contains supplementary material available at 10.1007/s00540-025-03526-6.

## Introduction

According to the growing recognition of the importance of frailty in surgical risk assessment [1–3], clinicians have continuously strived to identify patients at high risk for adverse postoperative outcomes. Traditional tools such as nutritional indicators and frailty indices are often challenging to implement in routine clinical practice due to limitations like the lack of standardized measurement tools, time constraint (e.g., obtaining laboratory results and expert assessments), and issues related to patient cooperation (e.g., completing questionnaires and undergoing physical exams).

In this context, bioimpedance analysis (BIA) has emerged as a simple yet effective way to measure frailty [4] and predict postoperative outcomes [5, 6]. BIA is a body composition analysis technique that measures the impedance to an alternating electrical current as it passes through body fluids and tissues. By analyzing resistance and reactance values, BIA calculates various parameters, including the phase angle (PA). PA, a key parameter derived from BIA, reflects the integrity of cell membranes and the distribution of body fluids. PA reflects hydration status and muscle mass and strength, which are closely linked to a patient’s nutritional status and frailty. A higher PA suggests intact cell membrane integrity (indicating better health), while a lower PA indicates decreased cell integrity (suggesting frailty). Unlike traditional frailty indices or nutritional markers, BIA-derived PA can be rapidly measured at the bedside, does not require patient cooperation, and provides immediate quantitative results with minimal evaluator bias. PA has been studied across various surgical fields as a predictor of clinical outcomes [5, 7–11]. However, there is still limited research on its prognostic significance in major abdominal surgery.

Therefore, we hypothesized that a lower preoperative PA would be significantly associated with adverse in-hospital postoperative outcomes in patients undergoing major abdominal surgery. In addition, the authors evaluated the association between PA and adverse outcomes occurring within 1 year after surgery.

### Methods

#### Study design

This prospective observational study was conducted at Samsung Medical Center, Seoul, Korea, between July 2019 and April 2021, in accordance with the Declaration of Helsinki. The study was approved by the Samsung Medical Center Institutional Review Board (SMC 2019-05-149-001) on June 17, 2019, and was registered in the Clinical Research Information Services (CRIS identifier KCT0004160) on July 24, 2019. The study was conducted in accordance with the principles of the Declaration of Helsinki. Written informed consent was obtained from each participant. We enrolled adult patients over 19 years old with American Society of Anesthesiologists Physical Status classification I to IV, scheduled for major abdominal surgery. Eligible surgeries were aortic, hepatobiliary, pancreatic surgery, and retroperitoneal mass excision via long midline laparotomy. The exclusion criteria were as follows: pregnant or lactating women, patients undergoing hemodialysis, those with implantable cardiac devices, individuals unable to cooperate with the study procedures, and patients expected to undergo minor abdominal surgery lasting less than 2 h. Patients undergoing hemodialysis were excluded due to potential variations in extracellular water content related to the timing of their dialysis sessions, which could influence PA measurements. In addition, patients with implantable cardiac devices were excluded based on the manufacturer’s guidance, as the electrical currents used in BIA measurement could potentially cause device malfunction. If the acquisition of the BIA test failed, the patient was considered a dropout.

#### BIA

**Fig. 1:**
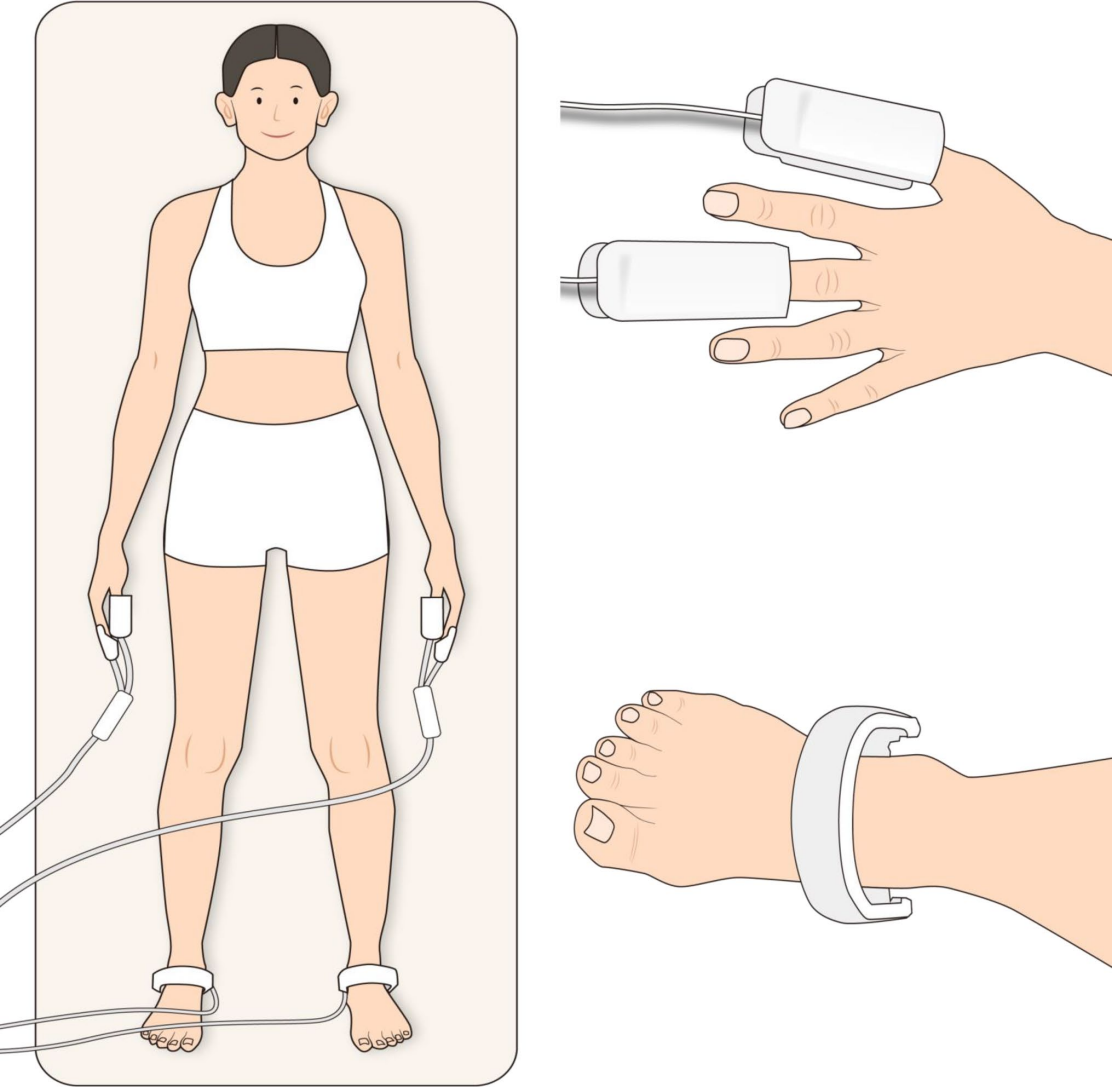
Schematic diagram of the bioelectrical impedance-analysis (BIA) measurement process. Patients lie in a supine position with four electrodes attached to the wrists and ankles, remaining still and relaxed during the measurement

The InBody S10® (InBody Co., Ltd., Seoul, Korea) is a multi-frequency (1, 5, 50, 250, 500, and 1000 kHz) bioelectrical impedance analyzer that automatically calculates the whole-body PA using impedance and reactance at 50 kHz. Patients were instructed to empty their bladder and to rest in bed without wearing any electronic devices or accessories for at least 30 min before the test. During the measurement, patients lay supine in a stable condition, with electrodes attached to the wrist and ankle in accordance with the manufacturer’s instructions (Fig. 1). The following data were collected and exported to an Excel file for analysis:

·PA (°) = (reactance/resistance) × (180°/π).

·ECWR = extracellular water/total body water.

·SMI = limb skeletal muscle mass/square of height.

·FFM = total body weight minus fat.

·BCM = total mass of all the cellular elements.

### Outcome measurement

Demographic data were extracted from electronic medical records. One trained investigator conducted BIA test and frailty test on the day before surgery (BIA_pre_). For the BIA test, we performed the two additional assessments: one immediately after the surgery (BIA_post_) and another 24 h later (BIA_POD1_). To compare with the PA measured by BIA, frailty was assessed using three different scales: Ganapathi indices, [12] modified frailty index (mFI), [13] and the Korean version of the Fatigue, Resistance, Ambulation, Illnesses, and Loss of weight scale (K-FRAIL). [14] The Ganapathi index is an objective indicator of frailty based on medical history and laboratory findings. The modified frailty index is a well-established tool for frailty assessment using functional status and medical history, whereas the K-FRAIL is focused on functional status. In addition, laboratory findings which reflect nutrition status, such as hemoglobin, blood cell count, and serum albumin, were collected. Prognostic nutritional index (PNI) was calculated using following formula: albumin level (g/L) + 0.005 × lymphocyte (count/µL) [15].

The occurrences of postoperative in-hospital complications, including all-cause mortality, MACCE, hemodynamic instability, acute kidney injury (AKI), postoperative pulmonary complications (PPC), delirium, ileus, and surgical complications were recorded. MACCE includes cardiac arrest, myocardial infarction, coronary revascularization, or stroke. Hemodynamic instability is defined as hypotension: systolic blood pressure < 85 mmHg or 20% decrease of preoperative value requiring treatment. AKI was defined according to the Kidney Disease Improving Global Outcomes criteria using creatinine concentration measured within 72 h postoperatively. PPC was defined as grade > 2 according to PPC scoring criteria described by Kroenke et al. in 1992 [16]. Surgical complications include wound complications, sepsis, surgical site bleeding requiring intervention, and reoperation.

Each patient was followed at 1 month, 3 month or 6 month, and 1 year postoperatively in the out-patient clinic. We collected data regarding adverse outcomes, such as major adverse cardiac and cerebrovascular events (MACCE), surgical complications and readmission for 1-year.

The primary outcome was a composite of in-hospital complications after major abdominal surgery. If a patient experienced one or more complications, it was counted as a single case. Secondary outcomes were a 1-year complication including all-cause mortality, MACCE, out-hospital surgical complications, and readmission within the year after surgery.

### Statistical analysis

Sample size calculation was performed based on clinical data from the authors’ institution. At Samsung Medical Center, in-hospital complications were observed in 30% of patients who underwent major abdominal surgery. According to data provided from the InBody manufacturer, the average PA of healthy Koreans in their ages of 60 s is 5.6, with the standard deviation (SD) of 0.6. When the average PA is 5.6, the postoperative complication rate is 30%, and when the PA is as low as 5 (mean value—standard deviation), the postoperative complication rate was expected to be 45%, by clinical assumption. Based on this, it was estimated that about 122 participants were required at a significance level of 5% and a power of 80%. Considering the dropout rate of 10%, we decided to enroll 136 participants.

The normality of distribution was assessed using a Shapiro–Wilk test for continuous variables. Normally distributed descriptive data was represented as mean ± SD, and skewed data was represented as median with interquartile range (IQR). Categorical data are presented as frequency with percentage. Correlations between frailty indices and BIA-derived variables were analyzed using Spearman’s rank correlation coefficient. To identify the risk factors for in-hospital complications, we performed logistic regression modeling using the variables of baseline characteristics and PA_pre_. The predictive value of BIA-derived variables was determined using receiver operating characteristic (ROC) curves.

All presented p values were considered statistically significant at <0.05. All statistical analysis was performed SPSS 29.0 (IBM SPSS, Inc, Armonk, NY).

## Results

**Fig. 2:**
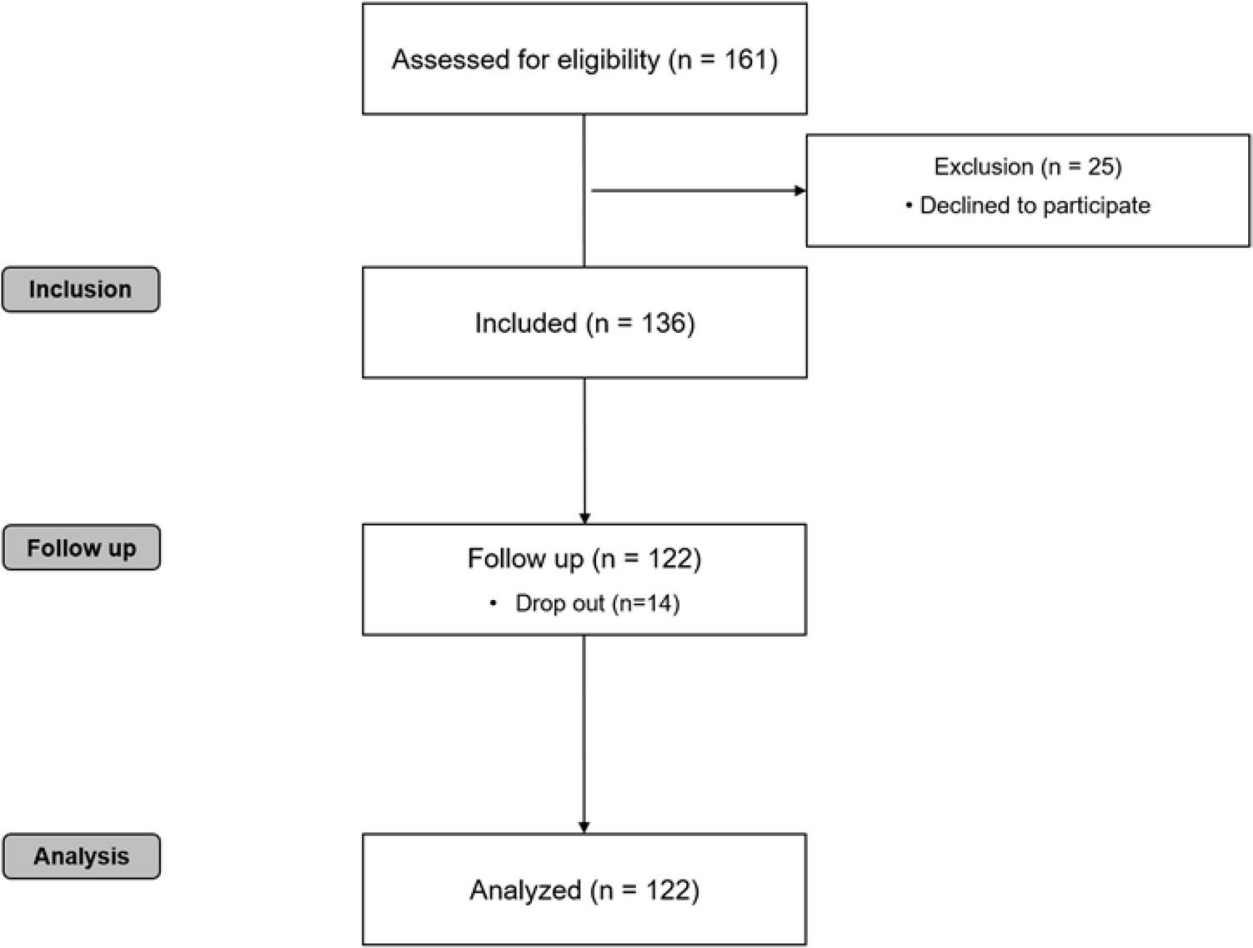
STROBE flow chart. STROBE, strengthening the reporting of observational studies in epidemiology flow chart

One hundred and sixty-one patients who underwent elective major abdominal surgery were assessed for eligibility. Of these, 136 participants enrolled, and 122 participants completed the study (Fig. 2). Table 1 presents the baseline characteristics according to PA_pre_. The median age of the participants was 64 years (IQR, 55–71), and 87 patients (71.3%) were men. Among the participants, 25 patients (20.5%) had a functional capacity of less than 4 metabolic equivalents, and 29 patients (23.8%) were found to have sarcopenia. A total of 110 participants were followed up for 1 year after surgery.

**Table 1:** Basic patient characteristics of the study population according to phase angle, Bbio-impedance analysis results, and frailty index according to baseline phase angle

Variables	Total (N = 122)	PA^a^ < 5.0 (n = 43)	PA^a^ ≥ 5.0 (n = 79)	*p* value
*Bio-impedance analyses at preoperative time point*				
Phase angle	5.5 [4.8, 6.1]	4.6 [4.2, 4.8]	5.8 [5.5, 6.4]	<0.001
ECWR	0.385 [0.380, 0.393]	0.395 [0.392, 0.399]	0.382 [0.377, 0.385]	<0.001
SMI	7.2 ± 1.1	6.4 ± 1.0	7.7 ± 0.9	<0.001
FFM	47.9 ± 8.9	42.2 ± 8.1	51.0 ± 7.8	<0.001
BCM	31.0 ± 6.0	26.9 ± 5.2	33.3 ± 5.1	<0.001
*Demographic variables*				
Age (year)	64.0 [55.0, 71.0]	68 [60.5, 75]	60 [53.5, 67]	<0.001
Height (cm)	165.3 ± 7.9	162.8 ± 8.8	166.7 ± 7.1	0.010
Weight (kg)	65.3 ± 10.6	61.1 ± 10.8	67.7 ± 9.9	0.001
BMI (kg·m^−2^)	24.0 ± 3.0	22.9 ± 3.0	24.3 ± 2.8	0.013
Gender (male: female)	87 (71.3):35 (28.7)	23 (53.5):20 (46.5)	64 (81.0):15 (19.0)	0.001
ASA-PS (I:II: III:IV)	6 (4.9):59 (48.4):55 (45.1):2 (1.6)	3 (3.8):44 (55.7):32 (40.5):0 (0.0)	3 (7.0):15 (34.9):23 (53.5):2 (4.7)	0.001
HTN	55 (45.1)	20 (46.5)	35 (44.3)	0.815
DM	25 (20.5)	10 (23.3)	15 (19.0)	0.577
Stroke	6 (4.9)	9 (20.9)	31 (39.2)	0.040
COPD	6 (4.9)	3 (7.0)	3 (3.8)	0.664
Chronic kidney disease	12 (9.8)	5 (11.6)	7 (8.9)	0.752
Heart failure	4 (3.3)	2 (4.7)	2 (2.5)	0.613
Past or current malignancy	58 (47.5)	19 (44.2)	39 (49.4)	0.584
*Type of surgery*				0.090
Aorta surgery	42 (34.4)	21 (48.8)	21 (26.6)	
Hepatectomy	45 (36.9)	14 (32.6)	31 (39.2)	
Retroperitoneal mass excision	23 (18.9)	5 (11.6)	18 (22.8)	
Other	12 (9.8)	3 (7.0)	9 (11.4)	
*Laboratory variables*				
Hemoglobin (g/dL)	13.6 [12.2, 14.4]	12.3 [10.9, 13.2]	14.1 [12.8, 14.8]	<0.001
Albumin (g/dL)	4.3 [4.0, 4.6]	4.0 [3.7, 4.4]	4.5 [4.2, 4.7]	<0.001
PNI	52.9 [48.2, 55.4]	48.2 [44.8, 53.9]	53.6 [51.3, 56.6]	<0.001
*Frailty index*				
Ganapathi index	1 [0, 1]	1 [0.5, 2]	0 [0, 1]	<0.001
mFI	2 [1, 3]	3 [1.5, 4]	2 [1, 3]	0.109
K-FRAIL	1 [1, 1]	2 [1, 3.5]	1 [1, 2]	0.007

Values are presented as frequency (%), mean ± SD, or median [IQR]

*ASA-PS* American Society of Anesthesiologists physical status, *BMI* body mass index, *BCM* body cell mass, *COPD* chronic obstructive pulmonary disease, *DM* diabetes mellitus, *ECWR* ECW ratio (extracellular water: total body water), *FFM* fat free mass, *IQR* interquartile range, *K-FRAIL* Korean version of the Fatigue, Resistance, Ambulation, Illness, and Loss of Weight scale, *mFI* modified frailty index, *PNI* prognostic nutritional index, *SD* standard deviation, *SMI* skeletal muscle index

^a^The PA was measured at preoperative time point

### Clinical outcomes

Approximately 41% of participants experienced one or more postoperative complications (Table 2). The primary outcome, in-hospital complication occurred more frequently in lower PA group (PA_pre_ < 5.0) than in the higher PA group (PA_pre_ ≥ 5.0). Twenty-three patients (53.5%) in the lower PA group presented in-hospital complications compared to 27 patients (34.2%) in the higher PA group. The most common complication was surgical complications (31.1%), followed by delirium (10.7%), and PPC (9.8%) and hemodynamic instability (9.8%). During the 1-year postoperative follow-up, 22 patients (20.0) experienced surgical complications, and 14 patients (12.3%) patients required readmission. MACCE was observed in 2 patiets in the higher PA group, and 1 patient in the lower PA group during the 1-year follow-up.

**Table 2:** Postoperative outcomes and complications

Variables	Total (N = 122)	PA^a^ < 5.0 (n = 43)	PA^a^ ≥ 5.0 (n = 79)	Relative risks (95% CI)	*p* value
In-hospital complications	50/122 (41.0)	23 (53.5)	27 (34.2)	1.6 (1.0 to 2.4)	0.038
MACCE or death	0/122 (0.0)	0 (0.0)	0 (0.0)	n/a	n/a
Hemodynamic instability	12/122 (9.8)	7 (16.3)	5 (6.3)	2.6 (0.9 to 7.6)	0.111
Acute kidney injury	9/122 (7.4)	5 (11.6)	4 (5.1)	2.3 (0.7 to 8.1)	0.276
Postoperative pulmonary complication	12/122 (9.8)	7 (16.3)	5 (6.3)	2.6 (0.9 to 9.6)	0.111
Delirium	13/122 (10.7)	7 (16.3)	6 (7.6)	2.1 (0.8 to 6.0)	0.217
Ileus	5/122 (4.1)	2 (4.7)	3 (3.58)	1.2 (0.2 to 7.1)	> 0.99
Surgical complication	38/122 (31.1)	17 (39.5)	21 (26.6)	1.5 (0.9 to 2.5)	0.140
Re-operation	8/122 (6.6)	3 (7.0)	5 (6.3)	1.1 (0.3 to 4.4)	> 0.99
*Postoperative outcomes*					
ICU admission	50/122 (41.0)	26 (60.5)	24 (30.4)	2.0 (1.3 to 3.0)	0.001
Length of postoperative stay in hospital, days	11 [10, 15]	11 [9.5, 15]	11 [10, 15.5]	Difference of medians, 0 (1 to 1)	0.996
1-year complications	23/110 (20.9)	7 (18.9)	16 (21.9)	0.8 (0.4 to 1.8)	0.715
Death	4/110 (3.6)	0 (0.0)	4 (5.4)	0.2 (0.0 to 3.7)	0.199
MACCE	3/110 (2.7)	1 (2.7)	2 (2.7)	0.9 (0.1 to 9.8)	> 0.99
Surgical complications	22/110 (20.0)	7 (18.9)	15 (20.5)	0.9 (0.4 to 1.9)	0.840
Readmission	14/110 (12.3)	5 (13.5)	9 (12.3)	1.0 (0.4 to 2.9)	> 0.99
Subgroup analysis according to gender^b^					
Male	Total (N = 88)	PA < 5.5 (n = 34)	PA ≥ 5.5 (n = 54)	Relative risks (95% CI)	
In-hospital complications	37/88 (42.0)	20/34 (58.8)	17/54 (31.5)	1.9 (1.2 to 3.0)	0.011
1-year complications	17/78 (21.8)	9/28 (32.1)	8/50 (16.0)	2.0 (0.9 to 4.6)	0.098
Female	Total (N = 32)	PA < 4.8 (n = 15)	PA ≥ 4.8 (n = 17)	Relative risks (95% CI)	
In-hospital complications	13/32 (40.6)	6/15 (60.0)	4/17 (23.5)	1.7 (0.6 to 4.9)	0.036
1-year complications	6/26 (18.8)	3/15 (20.0)	3/17 (17.6)	1.1 (0.3 to 4.8)	> 0.99

Values are presented as frequency (%) or median [IQR].

*MACCE* major adverse cardiac and cerebrovascular events; PA, phase angle

^a^The PA was measured at preoperative time point.

^b^The cut-off value (mean–standard deviation) was calculated based on data provided by the InBody manufacturer. The mean PA value was 6.0 ± 0.5 for males and 5.3 ± 0.5 for females. Accordingly, the cut-off values were determined as 5.5 for males and 4.8 for females.

#### BIA

**Fig. 3:**
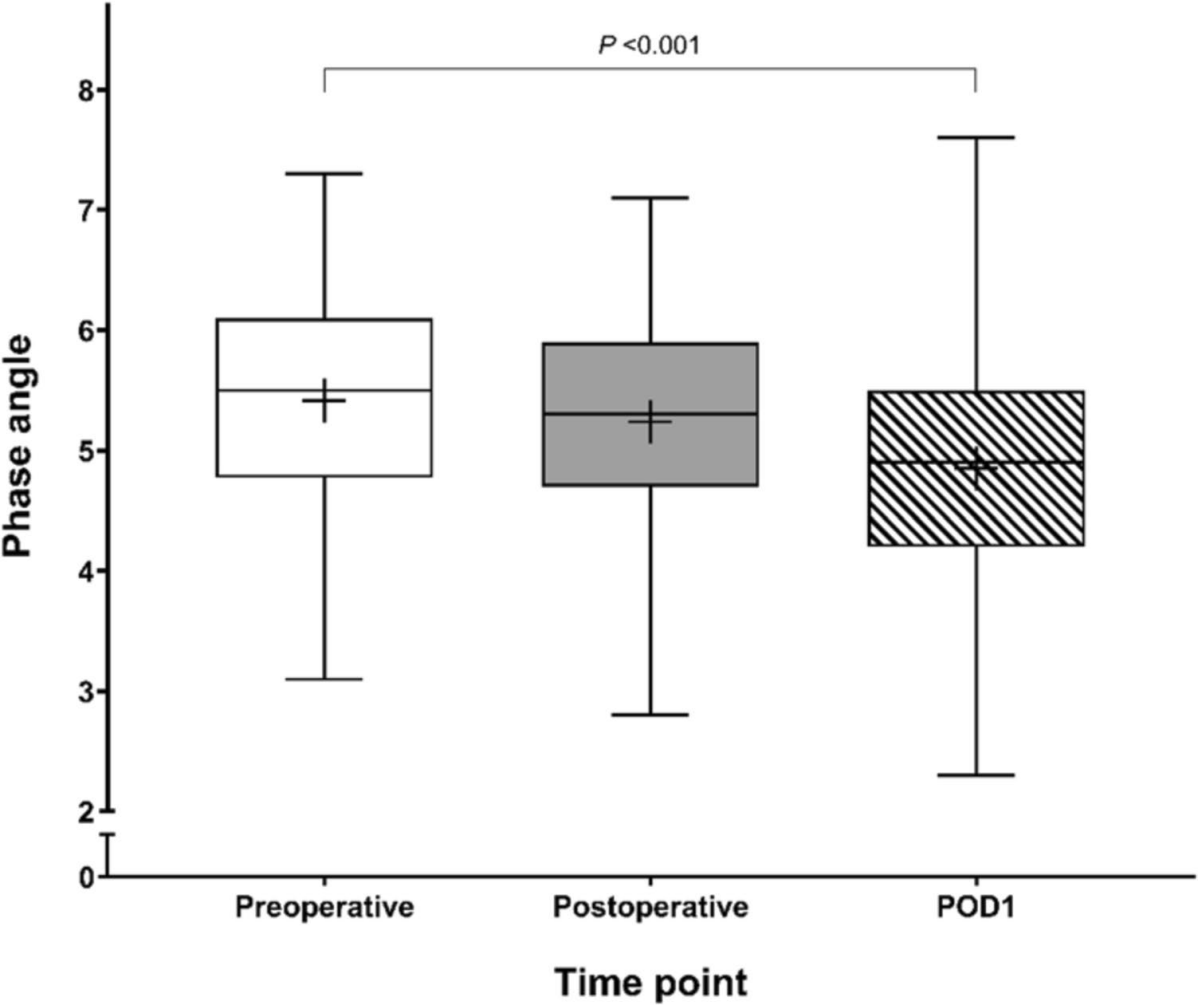
Box plot of phase angle at each time point. The horizontal line represents the median value, the box indicates the interquartile range (IQR), and the whiskers extend from the minimum to maximum values. The “+” symbol denotes the mean value. *POD* postoperative day

Figure 3 presents the perioperative trend of PA in patients undergoing major abdominal surgery. The median preoperative PA was 5.5 (IQR 4.8–6.1) and gradually decreased after surgery to 4.9 (IQR 4.2–5.5). According to the predetermined criteria, patients were divided into two groups based on a preoperative PA_pre_ threshold of 5.0. The ECWR_pre_ was higher in patients with lower PA_pre_ compared to those with higher PA_pre_ (*p* < 0.001). Conversely, SMI_pre_, FFM_pre_, and BCM_pre_ were lower in patients with lower PA_pre_ (all *p* < 0.001). Patients with lower PA_pre_ were significantly older and had a lower body mass index (BMI).

There was a significantly higher proportion of females in the group with lower PA_pre_. In laboratory variables, patients with lower PA_pre_ had lower hemoglobin and albumin levels, and their PNI was also lower, reflecting poorer nutritional status. Ganapathi index and K-FRAIL indicating frailty, showed that patients with lower PA_pre_ were frailer (Table 1).

##### BIA variables and clinical outcomes

Clinical outcomes are summarized in Table 2. In patients with lower PA_pre,_ the incidence of in-hospital complications was significantly higher (relative risk [RR], 1.6; 95% CI, 1.0 to 2.4; *p* = 0.038). Furthermore, ICU transfers occurred more frequently in these patients. However, PA_pre_ was not significantly associated with 1-year composite complications (*p* = 0.715).

We identified differences in BIA variables, demographic characteristics, and nutritional markers, such as hemoglobin, albumin, PNI, and frailty indices between patients who had in-hospital complications and those without (Supplementary Table 1). Based on these observed differences, 11 variables were selected and entered into a multivariable logistic regression model using a backward selection approach. In the final model, PA_pre_ and ASA-PS were significantly associated with in-hospital complications (PA_pre_: odds ratio [OR] 0.46; 95% CI, 0.27–0.79; *p* = 0.004; ASA-PS: OR, 2.72; 95% CI, 1.34–5.55; *p* = 0.006).

Given that PA values tend to differ by gender, we conducted separate analyses for males and females using gender-specific cut-off values (5.5 for males, 4.8 for females) to assess associations with in-hospital complications and 1-year composite complications. The subgroup analyses yielded results consistent with the combined analysis. For in-hospital complication, the relative risks were as follows: in males: RR = 1.9, *p* = 0.011; in females, RR = 1.7, *p* = 0.036. For 1-year composite complications, the relative risks were: in males, RR = 2.0, *p* = 0.098; in females, RR = 1.1, *p* > 0.99 (Table 2).

In the ROC analysis for prediction of in-hospital complications, the AUC was 0.662 (95% CI: 0.570–0.745, *p* = 0.001) for PA_pre_, 0.716 (95% CI: 0.627–0.794, *p* < 0.001) for PA_post_, and 0.722 (95% CI: 0.633–0.799, *p* < 0.001) for PA_POD1_ (Supplementary Fig. 1). The ROC analysis using PA_post_ and PA_POD1_ yielded increases in AUC and statistically significant (*p* = 0.013, 0.036, respectively). Each cut-off value of PA for the prediction of in-hospital complications was 5.7 for PA_pre_, 5.4 for PA_post_, and 5.0 for PA_POD1_, respectively.

##### Correlation analysis between BIA variables, and frailty and nutritional indices

The correlations of frailty and nutritional indices with BIA variables are shown in Supplementary Table 2. Patients with lower PA_pre_ had a higher an ECWR_pre_ (*p* < 0.001) and higher degrees of frailty, as measured by the Ganapathi index, mFI, and K-FRAIL. Lower PA_pre_ was also associated with older age, lower body weight, and lower hemoglobin concentration.

## Discussion

This prospective observational study suggests a possible association between preoperative PA (PA_pre_) and in-hospital complications in patients undergoing major abdominal surgery. Patients with a lower PA_pre_ appeared to have a higher incidence of in-hospital complications. While ASA-PS remains a well-established predictor of postoperative outcomes, a low PA_pre_ may also serve as a potential indicator for predicting in-hospital complications. PA measurements at the three time points were presumed to be robust predictors of in-hospital complications. However, neither PA_pre_, PA_post_, nor PA_POD1_ was significantly associated with 1-year composite complications.

BIA essentially evaluates body composition and volume status by analyzing the vectors of resistance and reactance as multi-frequency electric currents pass through the body. BIA provides various data, including ECWR, SMI, FFM, BCM, and PA. Previous studies have investigated whether ECWR reflects fluid administration during perioperative period [17, 18], and examined volume balance in cardiac disease [19] or dialysis patients [20]. BIA-derived PA offers intuitive information about a patient’s health status as a single parameter reflecting other body composition indicators, such as ECWR, SMI, and FFM. Prior research has shown its association with clinical prognosis after cardiac surgery and cancer surgery [5, 7–11]. While these previous studies primarily addressed impaired function [10, 11] or long term mortality [9] after surgery, the current study distinctively focused on the association between PA and short-term postoperative complications in patients undergoing major abdominal surgeries, examining this prospectively.

We established cut-off value of PA_pre_ for predicting in-hospital complication as 5.0 according to the manufacturer’s data, with the mean PA in Korean individuals in their 60 s being 5.6 and an SD of 0.6. However, ROC analysis suggested that the optimum cut-off value of PA_pre_ was 5.7, which was higher than expected. In a study of patients with gastrointestinal and hepatobiliary-pancreatic cancer, the cutoff for longer postoperative critical care was 4.4 in men and 4.0 in women [9]. In cardiac surgery, the PA cut-off for predicting mortality at 12 months was 4.5 [7], which was relatively lower than that of current study. This discrepancy is possibly attributed to the fact that this study aimed to investigate the association of PA and composite of in-hospital complications, not critical events. Similarly, our previous work proposed the cut-off value for in-hospital complications in patients undergoing off-pump coronary artery bypass grafting as 6.0, which was similar in this study.

PA may serve as a prognostic predictor for short-term postoperative outcomes, as it facilitates the early identification of high-risk patients with vulnerable physiologic reserves. Consistent with previous literature [6–8, 21–24], the results of the current study confirmed that PA was significantly associated with parameters of frailty (age, Ganapathi index, mFI, K-FRAIL) as well as nutritional status indicators (weight, hemoglobin, albumin, PNI). In contrast to other time-constrained frailty indices, PA can be readily quantified by a BIA device at the bedside and can be easily integrated into everyday clinical practice without incurring additional medical costs and requiring extensive patient cooperation. Specifically, for patients in the acute phase of postoperative recovery who may be unable to complete performance-based frailty assessments, PA can still provide valuable information about their health status.

The additional clinical role of preoperative PA, beyond its prognostic value, lies in its potential to guide supportive care during the perioperative period. While studies focusing on surgical patients are limited, previous reports suggest that nutritional and exercise interventions can improve PA, body composition, and functional status [25–28]. Ideally, PA should be assessed early when surgery is planned, followed by tailored prehabilitation programs. Repeated PA assessments can help monitor patient progress, guide interventions, and support recovery. However, the appropriate timeline or intensity of physical and nutritional prehabilitation may depend on the individual health status, treatment goals, and response to interventions. Further research is needed in this area.

For anesthesiologists, PA_pre_ may help identify patients at higher risk of complications, allowing for better allocation of resources, close monitoring with more tight target values, and timely interventions.

Interestingly, PA measured postoperatively (PA_post_ and PA_POD1_) demonstrated greater discriminatory power than PA_pre_. Because our study was not designed to compare PA_pre_ with PA _post_ or PA_POD1_, it is difficult to conclude which time point is superior.

However, our results suggest that PA measurements taken closer to or after surgery also have clinical significance in predicting in-hospital complications. While PA_pre_ primarily reflects baseline physiologic and nutritional status, PA_post_ and PA _POD1_ may incorporate perioperative dynamics such as surgical complexity, intraoperative insults, and perioperative fluid management, which are particularly relevant in major abdominal surgery. The cut-off values for predicting in-hospital complications were 5.7 for PA_pre_, 5.4 for PA_post_, and 5.0 for PA _POD1_. The progressive decline in these thresholds over time likely reflects the cumulative impact of surgical trauma, metabolic stress, inflammatory responses, and perioperative fluid shifts, all of which contribute to postoperative physiologic changes. Moreover, the potential utility of PA_post_ and PA_POD1_ in situations where preoperative assessments are not feasible, such as emergency surgeries, suggests that perioperative PA measurements may serve as an adjunctive tool. These findings suggest that perioperative PA measurements, which may change in response to surgical stress and perioperative management, can complement preoperative assessments to enhance risk stratification. However, further research is needed to elucidate the mechanisms driving perioperative PA changes and to validate its prognostic utility across broader surgical populations. Moreover, while PA demonstrated prognostic value in our study, its predictive accuracy was within the range generally considered low to moderate. Therefore, PA alone is not sufficient for clinical decision-making but may still provide meaningful information when used alongside established prognostic factors. Further studies are needed to validate the clinical utility of PA in broader surgical populations and to explore its integration with established prognostic factor.

The result of the current study should be interpreted with the following limitations. First, the study cohorts consisted of a relatively heterogeneous group of participants. Most of the participants were diagnosed with either malignancy (68/122, 55.8%) or aortopathy (42/122, 34.4%), and the contrasting characteristics of these two groups may have acted as potential confounders. Patients with malignancy were affected by factors such as cancer stage, type (aggressiveness and pathology) and the need for chemotherapy, whereas patients with aortopathy were cured completely by surgery alone without the need of additional treatments. In addition, patients with conditions that could cause fluid retention, such as chronic kidney disease (CKD) and heart failure (HF), were not excluded from the study. While excluding dialysis patients with severe CKD likely mitigated major concerns, the inclusion of HF patients may still represent a limitation. Second, BIA requires stable patient conditions and an environment free from interference by external electronic devices to yield accurate results. However, the perioperative environment can be less stable, with factors such as hypothermia, skin contamination from antiseptic solutions, and the presence of surgical drains potentially affecting PA accuracy. To mitigate these issues, we ensured that preoperative PA measurements were conducted under consistent and controlled conditions by a single, experienced researcher. Third, this study included only patients undergoing elective surgery, excluding those with unstable vital signs. Accurate acquisition of BIA variables necessitates detaching monitoring devices, which restricts its practicality in emergency settings where continuous monitoring is crucial. Fourth, PA is influenced by race, gender, and age. However, due to the lack of established reference values specific to our study population, we arbitrarily used a cut-off value of 5.0 to stratify patients for the analysis of postoperative complications. Future studies should consider incorporating standardized PA values that account for age and gender to enhance the accuracy and applicability of PA as a prognostic marker. Last, the total cases of MACCE and readmission in our study were small. Therefore, the study may have been underpowered to detect the association between PA and 1-year outcomes.

In conclusion, low preoperative PA was associated with adverse postoperative outcomes after major abdominal surgery. Our results indicate that PA can be a reliable prognostic factor to predict clinical outcomes in patients undergoing major abdominal surgery, serving as an alternative surrogate to frailty indices and nutritional markers.

## Supplementary Information

Below is the link to the electronic supplementary material.

Supplementary file1 (DOCX 72 KB)

## Data Availability

The authors confirm that the data supporting the findings of this study are included in this article and its supplementary materials. Individual participant data and additional data are available from the corresponding author, Jeong-Jin Min, upon request.
