# Supplementary material for: Phase angle as a prognostic factor for postoperative outcomes in major abdominal surgery: a single-center prospective observational study
Source: J Anesth. 2025 Jun 13;39(5):750–9. doi: 10.1007/s00540-025-03526-6 (PMC12464096; doi:10.1007/s00540-025-03526-6)

**Supplementary Table 1** Basic patient characteristics, Bio-impedance analysis results, and frailty index according to complications within hospitalization after surgery

| Variables | Total (N = 122) | Complications (n = 48) | No complications (n = 74) | *P* value |
| --- | --- | --- | --- | --- |
| **Bio-impedance analyses, preoperative** | | | | |
| Phase angle | 5.4 ± 0.9 | 5.1 ± 0.9 | 5.6 ± 0.8 | < 0.001 |
| ECWR | 0.386 ± 0.010 | 0.389 ± 0.010 | 0.384 ± 0.009 | < 0.001 |
| SMI | 7.2 ± 1.1 | 7.0 ± 1.2 | 7.3 ± 1.0 | 0.001 |
| FFM | 47.9 ± 8.9 | 46.2 ± 9.0 | 49.0 ± 8.7 | < 0.001 |
| BCM | 31.0 ± 6.0 | 29.8 ± 6.1 | 31.8 ± 5.8 | < 0.001 |
| **Demographic variables** |  |  |  |  |
| Age (yr) | 64.0 [55.0, 71.0] | 69.5 [60.5, 76.0] | 60.0 [51.0, 67.0] | < 0.001 |
| Height (cm) | 166.0 [160, 170.0] | 165.3 [159.5, 168.7] | 166.8 [160.3, 171.0] | 0.063 |
| Weight (kg) | 65.3 ± 10.6 | 63.7 ± 10.9 | 66.3 ± 10.3 | < 0.001 |
| BMI (kg·m^-2^) | 24.0 ± 3.0 | 23.9 ± 3.3 | 24.0 ± 2.8 | 0.814 |
| Gender (Male: Female) | 87 (71.3): 35 (28.7) | 36 (75.0): 12 (25.0) | 51 (68.9): 23 (31.1) | 0.046 |
| ASA-PS (Ⅰ: Ⅱ: Ⅲ: Ⅳ) | 6 (4.9): 59 (48.4): 55 (45.1): 2 (1.6) | 6 (8.3): 40(55.6): 26 (25.0): 0 (0.0) | 0 (0.0): 19 (38.0): 29 (58.0): 2 (4.0) | 0.004 |
| HTN | 55 (45.1) | 22 (45.8) | 33 (44.6) | 0.392 |
| DM | 25 (20.5) | 13 (17.6) | 12 (25.0) | 0.588 |
| Stroke | 6 (4.9) | 2 (2.7) | 4 (8.3) | > 0.99 |
| COPD | 6 (4.9) | 2 (2.7) | 4 (8.3) | > 0.99 |
| Chronic kidney disease | 12 (9.8) | 7 (9.5) | 5 (10.4) | 0.233 |
| Heart failure | 4 (3.3) | 1 (1.4) | 3 (6.3) | 0.523 |
| Past or current malignancy | 58 (47.5) | 38 (51.4) | 20 (41.7) | 0.260 |
| **Laboratory variables** | | | | |
| Hemoglobin (g/dL) | 13.6 [12.2, 14.4] | 12.9 [11.4, 14.5] | 13.7 [12.5, 14.4] | 0.026 |
| Albumin (g/dL) | 4.3 [4.0, 4.6] | 4.2 [3.9. 4.5] | 4.4 [4.2, 4.6] | 0.001 |
| PNI | 52.9 [48.2, 55.4] | 52.4 [45.1, 55.1] | 52.4 [50.6, 55.4] | 0.001 |
| **Frailty index** | | | | |
| Ganapathi index | 1 [0, 1] | 1 [0, 2] | 0 [0, 1] | 0.005 |
| mFI | 2 [1, 3] | 3 [2, 4] | 2 [1, 3] | 0.579 |
| K-FRAIL | 1 [1, 1] | 2 [1, 4] | 1 [1, 2] | 0.026 |

Values are presented as frequency (%), mean ± SD, or median [IQR]

*ASA-PS* American Society of Anesthesiologists physical status, *BMI* body mass index, *BCM* body cell mass, *COPD* chronic obstructive pulmonary disease, *DM* diabetes mellitus, *ECWR* ECW ratio (extracellular water: total body water), *FFM* fat free mass, *IQR* interquartile range, *K-FRAIL* Korean version of the Fatigue, Resistance, Ambulation, Illness, and Loss of Weight scale, *mFI* modified frailty index, *PNI* prognostic nutritional index, *SD* standard deviation, *SMI* skeletal muscle index

**Supplementary Table 2** Correlations between BIA Variables, frailty indices, and nutritional markers

|  | Phase angle | ECWR | Age | Weight | Hemoglobin | Albumin | PNI | Ganapathi index | mFI | K-FRAIL |
| --- | --- | --- | --- | --- | --- | --- | --- | --- | --- | --- |
| Phase angle | - | - 0.906^**^ | -0.316^**^ | 0.391^**^ | 0.465^**^ | 0.451^**^ | 0.425^**^ | -0.475^**^ | -0.195^*^ | -0.360^**^ |
| ECWR | - 0.906^**^ | - | 0.319^**^ | -0.296^**^ | -0.475^**^ | -0.440^**^ | -0.394^**^ | 0.472^**^ | 0.248^**^ | 0.403^**^ |
| Age | -0.316^**^ | 0.319^**^ | - | -0.176 | -0.141 | -0.326^**^ | -0.197^*^ | 0.480^**^ | 0.321^**^ | 0.175 |
| Weight | 0.391^**^ | -0.296^**^ | -0.176 | - | 0.171^*^ | -0.002 | 0.033 | -0.151 | 0.005 | -0.255^**^ |
| Hemoglobin | 0.465^**^ | -0.475^**^ | -0.141 | 0.181^*^ | - | 0.455^**^ | 0.542^**^ | -0.630^**^ | -0.085 | -0.228^*^ |
| Albumin | 0.451^**^ | -0.440^**^ | -0.326^**^ | -0.002 | 0.455^**^ | - | 0.767^**^ | -0.416^**^ | -0.140 | -0.173 |
| PNI | 0.425^**^ | -0.394^**^ | -0.197^*^ | 0.033 | 0.542^**^ | 0.767^**^ | - | -0.416^**^ | -0.140 | -0.173 |
| Ganapathi index | −0.475^**^ | 0.472^**^ | 0.480^**^ | -0.151 | -0.630^**^ | -0.416^**^ | -0.427^**^ | - | 0.321^**^ | 0.307^**^ |
| mFI | −0.195^*^ | 0.248^**^ | 0.321^**^ | 0.005 | -0.085 | -0.140 | 0.001 | 0.321^**^ | - | 0.311^**^ |
| K-FRAIL | −0.360^**^ | 0.403^**^ | 0.175 | -0.255^**^ | -0.228^*^ | -0.173 | -0.179^*^ | 0.307^**^ | 0.311^**^ | - |

*BIA* bio-impedance analysis, *ECWR* extracellular water ratio, *FFM* fat-free mass, *K-FRAIL* Korean version of the Fatigue, Resistance, Ambulation, Illness, and Loss of Weight scale, *mFI* modified frailty index, *PNI* prognostic nutritional index, *SMI* skeletal muscle index, *TBW* total body water

^*^*P* < 0.05 ^**^*P* < 0.01

**Supplementary Fig. 1** Receiver operating characteristic analysis of phase angle at three time points for prediction of in-hospital complications


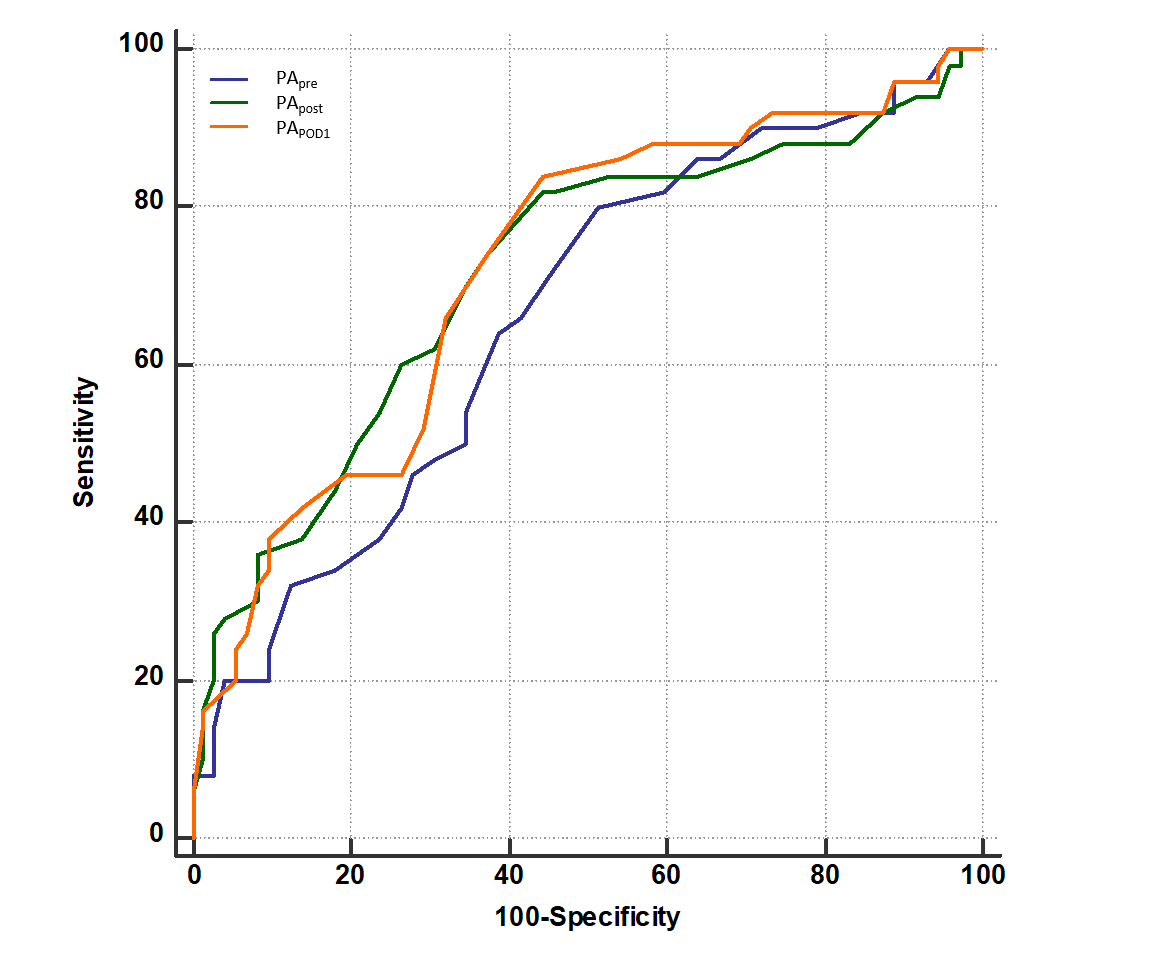

Supplement: Supplementary file 1 — Supplementary file1 (DOCX 72 KB) [file 540_2025_3526_MOESM1_ESM.docx]
